# Whole-Exome Sequencing Identifies a Novel Genotype-Phenotype Correlation in the Entactin Domain of the Known Deafness Gene *TECTA*

**DOI:** 10.1371/journal.pone.0097040

**Published:** 2014-05-09

**Authors:** Byung Yoon Choi, Jiwoong Kim, Juyong Chung, Ah Reum Kim, Sue Jean Mun, Seong Il Kang, Sang-Heon Lee, Namshin Kim, Seung-Ha Oh

**Affiliations:** 1 Department of Otorhinolaryngology, Seoul National University Bundang Hospital, Seongnam, South Korea; 2 Korean Bioinformation center, Korea Research Institute of Bioscience and Biotechnology (KRIBB), Daejeon, South Korea; 3 Department of Otolaryngology, Ajou University School of Medicine, Suwon, South Korea; 4 Department of Otorhinolaryngology, Seoul national University College of Medicine, Seoul, South Korea; 5 Department of Bioinformatics, University of Science and Technology, Daejeon, South Korea; Oslo University Hospital, Norway

## Abstract

Postlingual progressive hearing loss, affecting primarily the high frequencies, is the clinical finding in most cases of autosomal dominant nonsyndromic hearing loss (ADNSHL). The molecular genetic etiology of ADNSHL is extremely heterogeneous. We applied whole-exome sequencing to reveal the genetic etiology of high-frequency hearing loss in a mid-sized Korean family without any prior linkage data. Whole-exome sequencing of four family members (two affected and two unaffected), together with our filtering strategy based on comprehensive bioinformatics analyses, identified 21 potential pathogenic candidates. Sanger validation of an additional five family members excluded 20 variants, leaving only one novel variant, *TECTA* c.710C>T (p.T237I), as the strongest candidate. This variant resides in the entactin (ENT) domain and co-segregated perfectly with non-progressive high-frequency hearing loss in the family. It was absent among 700 ethnically matched control chromosomes, and the T237 residue is conserved among species, which supports its pathogenicity. Interestingly, this finding contrasted with a previously proposed genotype-phenotype correlation in which variants of the ENT domain of *TECTA* were associated with mid-frequency hearing loss. Based upon what we observed, we propose a novel “genotype to phenotype” correlation in the ENT domain of *TECTA*. Our results shed light on another important application of whole-exome sequencing: the establishment of a novel genotype-phenotype in the molecular genetic diagnosis of autosomal dominant hearing loss.

## Introduction

Postlingual progressive hearing loss, primarily affecting higher frequencies, is the clinical finding in most cases of autosomal dominant nonsyndromic hearing loss (ADNSHL) [1]. ADNSHL is extremely heterogeneous. To date, 64 loci and 27 autosomal genes have been identified (http://hereditaryhearingloss.org). Mutation screening is rarely offered to patients, owing to the extreme clinical and genetic heterogeneity of ADNSHL. Regarding this heterogeneity, *TECTA* has been identified as the causative gene for DFNA8/12 [2], [3] as well as DFNB21 [4]. Alpha-tectorin, a major non-collagenous component of the tectorial membrane, is encoded by the *TECTA* gene located on human chromosome 11q22-24 [3], [5], [6]. Alpha-tectorin has several functional domains: the entactin (ENT)-like domain, the large zonadhesin region containing four von Willebrand factor–like type D domains, and the zona pellucida domain [5], [7], [8]. Autosomal dominant missense mutations in *TECTA* give rise to various hearing loss phenotypes, depending on the protein domains in which the mutations reside, in contrast with autosomal recessive mutations that show similar phenotypes [3], [9]-[20].

*TECTA* is a highly polymorphic gene [10], and functional analyses of the variants of *TECTA* are not achieved easily, except in limited cases [21], [22]. Therefore, confirming that the detected variant of *TECTA* truly accounts for hearing loss and excluding other genes that may be responsible for the phenotype are extremely difficult without proper linkage data. However, many families are too small for linkage studies to be undertaken. To overcome this limitation, researchers have proposed audioprofiling as a method of categorizing phenotypic data to make genotypic correlations [18]. Recent advances in DNA enrichment, followed by next-generation sequencing technologies, have allowed the rapid and cost-effective sequencing of all the exons in the genome, to identify the causative alleles responsible for Mendelian disorders [23], [24]. This technique is expected to be useful for identifying the molecular genetic etiology of deafness, because most genetic hearing loss is caused by monogenic lesions [25]. Indeed, numerous new causative genes for hearing loss have recently been identified successfully using next-generation sequencing [26]-[33].

We applied whole-exome sequencing to reveal the molecular genetic etiology of high-frequency hearing loss segregating in one mid-sized Korean family, SNUBH18 (Seoul National University Bundang Hospital 18). In this study, whole-exome sequencing and further sequence analyses clearly identified a genetic defect underlying the hearing loss within the ENT domain of *TECTA*, defying a conventional genotype-phenotype correlation and establishing a diverse correlation in the ENT domain of the gene.

## Methods

### Human subjects

**Figure 1:**
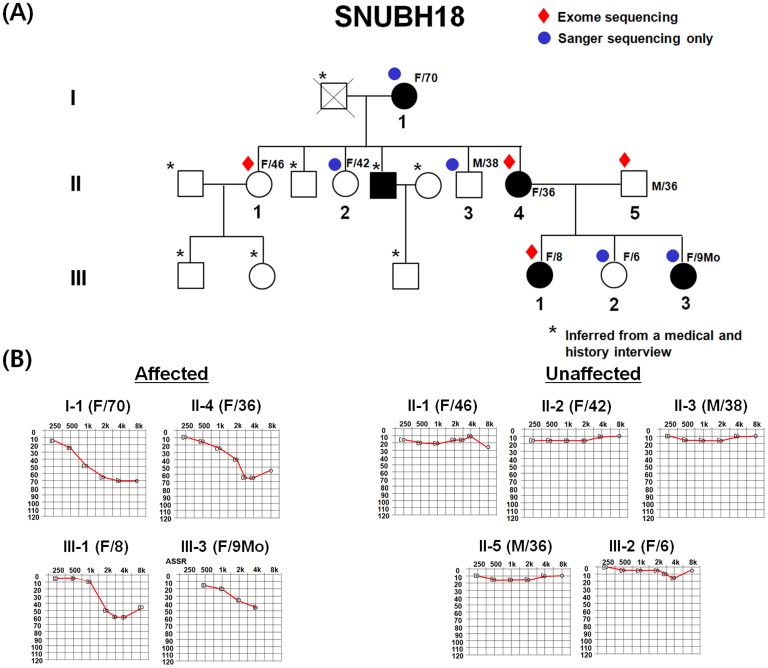
Pedigree and pure tone audiograms of the family SNUBH18. (A) Whole exome sequencing was performed for two affected (III-1 and II-4) and two unaffected individuals (II-5 and II-1) (red diamond). An additional five individuals (two affected and three unaffected) (blue) were recruited for Sanger validation and further filtering. (B) Individuals I-1, II-4, III-1, and III-3 showed typical high frequency hearing loss, while the others showed a normal hearing threshold over all frequencies.

All procedures in this study were approved by the institutional review boards at Seoul National University Hospital (IRBY-H-0905-041-281) and Seoul National University Bundang Hospital (SNUBH: IRB-B-1007-105-402). Written informed consent was obtained from all individuals or guardians. The pedigree comprised 16 individuals, nine of whom were willing to join the study. Nine members (SB18 I-1, II-1, 2, 3, 4, 5, III-1, 2 and 3) from the SNUBH18 family were identified and evaluated at the Seoul National University Hospital and SNUBH for this study (see Fig. 1). Phenotype evaluations included medical and developmental history interviews, physical examinations, and pure-tone audiometry.

### Audiometric evaluation

Pure-tone audiometry with air and bone conduction at frequencies ranging from 250–8000 Hz was carried out on the recruited subjects, according to standard protocols. The hearing loss range was described as follows, depending on the pure-tone audiometry results: low frequency, 250–500 Hz; mid frequency, 1–2 kHz; and high frequency, 4–8 kHz [34]. First-visit pure-tone audiograms for eight individuals and the auditory steady state response (ASSR) results for a 9-month-old child (SB18 III-3) are shown in Fig. 1. We calculated the mean level of hearing loss over all frequencies and at each frequency based upon the pure tone audiograms. We also calculated the mean hearing level of low frequencies, mid frequencies, and high frequencies. Temporal bone computed tomography was obtained from subjects II-4 and III-1 to identify any inner ear anomalies related to hearing loss in this family.

### Whole-exome sequencing

DNA from four of the nine recruited subjects (two affected and two unaffected) were selected for a commercial whole-exome sequencing service (Otogenetics, Norcross, GA, USA; Fig. 1). In total, 32–69 million short reads (90-bp paired-end reads) were obtained via whole-exome sequencing. More than 85% of the target exon regions were covered by at least five sequence reads. Alignment of the exome sequences was performed using the Burrows Wheeler Aligner (http://bio-bwa.sourceforge.net/). Picard software (http://picard.sourceforge.net/) was used to remove duplicates, and mate information was corrected using the FixMateInformation and MarkDuplicates modules. Regions around short indels were realigned using IndelRealigner, and base quality scores were recalibrated using the CountCovariates and TableRecalibration modules of the Genome Analysis Toolkit [35].

SNPs and short indels were identified and filtered using the UnifiedGenotyper and VariantFiltration modules, and annotated using the VariantAnnotator module of the Genome Analysis Toolkit. Finally, all variants were compared and tagged using the Single Nucleotide Polymorphism database (dbSNP build 138) and an in-house database, an independent cohort comprising 54 normal Korean individuals. Multigenome conservation scores (phyloP) across 46 vertebrate species from the University of California, Santa Cruz genome browser (http://genome.ucsc.edu) were also used to find variants of the conserved chromosome region. The detected novel variants have been submitted to the publicly available Leiden Open Variation Database repository (https://grenada.lumc.nl/LOVD2/Usher_montpellier/variants.php?select_db=TECTA&action=view&view=0003658). We utilized another cohort comprising 280 normal Korean control subjects to exclude rare *TECTA* variants.

## Results

### Auditory phenotype

Pure-tone audiograms of four affected individuals showed bilateral, moderate, symmetrical, and stable sensorineural hearing loss, most significantly that involving high frequencies (Fig. 1). The mean ± standard deviation (SD) levels of hearing impairment at 250, 500, 1000, 2000, 4000, and 8000 Hz from three older subjects were described (Table 1). The mean threshold level of 4 kHz was the lowest among the six frequencies, followed by that of 8 kHz, most significantly involving high frequencies. This indicated that the hearing impairment was most severe at high frequencies, followed by mid frequencies and then low frequencies. Insufficient follow-up time precluded serial audiograms in the affected subjects to detect any progression of hearing loss. Therefore, we compared the audiograms among the three affected SB18 subjects with their ages being roughly three decades apart. Progression of hearing loss did not seem to be significant, because the average aggravation rate (0.64 dB/yr) did not exceed 1 dB/yr for 1 kHz, which showed the most prominent aggravation among those at other frequencies in the affected subjects. The aggravation rates for 0.5 kHz, 2 kHz, and 4 kHz were 0.28, 0.24, and 0.20 dB/yr, respectively. Subjects II-4 and I-1 recollected that they noticed mild hearing loss and abnormal pronunciation during their early teens. They denied experiencing rapid progression of hearing loss. Subject III-1 (F/8yr) did not complain of hearing loss, however her pronunciation was mildly abnormal. All subjects denied any exposure to risk factors such as drugs or loud noise. No syndromic features were detected in the physical examination. Subject II-4 was reluctant to wear a hearing aid; however, subject III-2 (F/6yr) had begun wearing a hearing aid.

**Table 1:** The mean ± standard deviation (SD) level of hearing impairment at 250, 500, 1000, 2000, 4000, and 8000 Hz from three older subjects.

	Hearing threshold (dB)(mean + standard deviation)	
Frequency (Hz)	Right	Left
*Low frequency*		
250Hz	10.0 ± 5.0	11.7 ± 5.7
500Hz	15.0 ± 10.0	18.3 ± 11.5
	*12.5 ± 7.5*	*15.0 ± 8.9*
*Mid frequency*		
1000Hz	28.3 ± 20.2	31.7 ± 20.2
2000Hz	51.6 ± 12.6	53.3 ± 10.4
	*40 ± 19.7*	*42.5 ± 18.6*
*High frequency*		
4000Hz	65.0 ± 5.0	65.0 ± 8.7
8000Hz	56.7 ± 12.6	55.0 ± 15.0
	*60.8 ± 9.7*	*60 ± 12.2*
***Total (all frequencies)***	**37 ± 23.9**	**39.2 ± 23.1**

### Exome sequencing data analysis

**Figure 2:**
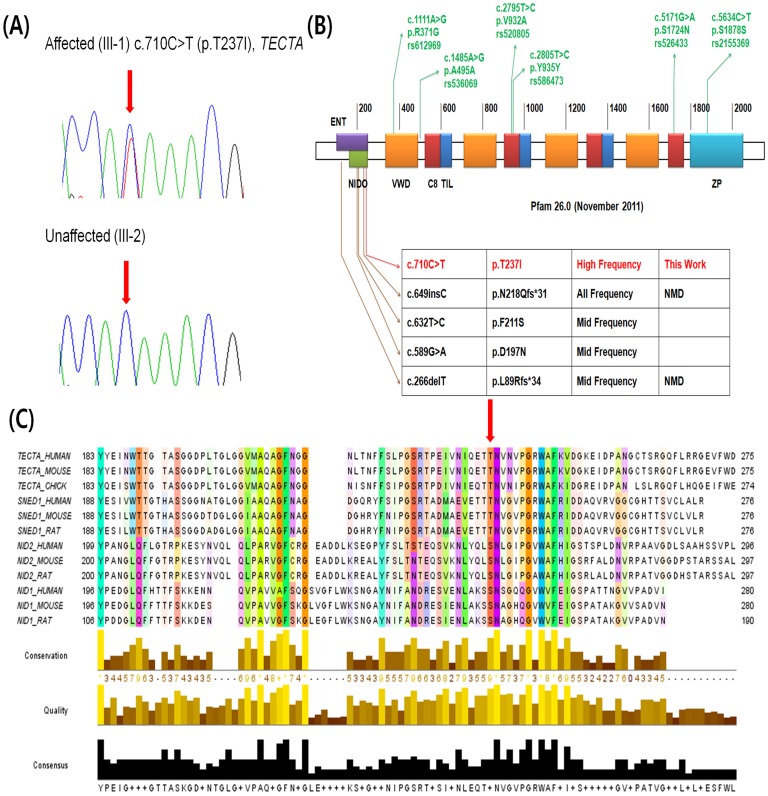
p.T237I variant of *TECTA* in SNUBH18. (A) Sanger sequencing traces of the p.T237I heterozygote and the wildtype. (B) Sequence variants related to hearing loss in the ENT domain of the *TECTA* gene. *TECTA* has five protein domains (NIDO, VWD, C8, TIL and ZP). To date, two missense and two frameshift indel variants related to mid- and all-frequency hearing loss have been discovered in the ENT domain. In contrast, p.T237I in the ENT domain is associated with high-frequency hearing loss. (C) Protein conservation of NIDO proteins among vertebrate species. p.T237 is well-conserved with threonine in Tecta and serine in NIDO proteins.

Ninety percent of the reads were mapped onto a human reference genome, achieving 20.7–45.4× coverage (44% on target reads; Table S1). Each individual contained 259–431 novel single-nucleotide polymorphisms (SNPs) and 3–28 novel short indels, which were used to detect novel pathogenic mutations in this family. Of these variants, approximately 65% of the novel SNPs were predicted, using SIFT software, to be deleterious mutations [36]. In total, 70 SNPs and three indels were selected as primary candidate alleles inherited by the affected daughter (III-1) from the affected mother (II-4), and which did not exist in individuals II-1 and II-5. Next, we prioritized 21 candidates (20 SNPs and one insertion) determined as deleterious using the following criteria: a phyloP score of at least 1.5 and a prediction of “damaging” by the SIFT software. Additional software analyses (Polyphen-2 [37], MutationTaster [38], and MutationAssessor [39]) gave similar overall predictions based on their own parameters. Finally, Sanger validation of an additional five individuals (I-1, II-2, II-3, III-2, and III-3; Fig. 1) and a control study from two independent cohorts were adequate for identifying one causative SNP, *TECTA* c.710 C>T (p.T237I) (Table 1). This *TECTA* variant was absent in a control population of 280 Koreans (560 chromosomes). The *TECTA* gene in this family harbors four intronic SNPs (rs681311, rs504626, rs2186747, and rs543577), as well as three missense SNPs (rs612969, rs520805, and rs526433) and three silent SNPs (rs536069, rs586473, and rs2155369; Table S2). These 10 SNPs, which apparently do not affect hearing loss, were also present in our in-house database. The sequence variant c.710 C>T resides in the nidogen (NIDO)-like domain, formerly known as the ENT domain (Table S3) (Fig. 2B). This variant (p.T237I) on the ENT domain co-segregated with high-frequency hearing loss in this study, as shown in Fig. 2A.

### Protein structure homology modeling and conservation

The protein structure of *TECTA* is not yet known, but we performed homology modeling using the *E. coli* chain A, L-fucose isomerase (1FUI_A). However, insufficient information was available due to the large differences between the human and *E.coli* protein sequences. A contact-map inferred from the DISTIL utility (http://distill.ucd.ie) predicted that the mutant residue would be exposed to the surface when the residue was converted from threonine to isoleucine. In contrast, the wild type threonine residue was embedded in the structure, suggesting a significant effect of p.T237I on protein structure (Fig. S1). Amino acid conservation within the NIDO domain indicated that the p.T237 residue was well-conserved among vertebrate species, with threonine (TECTA and SNED1 proteins) and serine (NID1 and NID2 proteins) polar, uncharged side chains (Fig. 2).

## Discussion

The majority of missense mutations that cause hearing loss related to *TECTA* reside in the zona pellucida and zonadhesin domains of the gene [16]. Previous studies have discovered only four probable pathogenic mutations within the ENT domain (Fig. 2). Two truncation mutations, c.266delT (p.L89Rfs*34) and c.649insC (p.N218Qfs*31), have been found in autosomal recessive nonsyndromic hearing loss. These two frameshift recessive mutations are associated with mid-frequency and all-frequency sensorineural hearing loss, respectively [17], [40]. The autosomal dominant missense mutations c.632T>C (p.F211S) and c.589G>A (p.D197N) in the NIDO domain have been clearly associated with mid-frequency sensorineural hearing loss [16], suggesting a genotype-phenotype correlation between mutations in the NIDO domain of *TECTA* and mid-frequency sensorineural hearing loss.

However, this study revealed that carriers of the variant c.710 C>T (p.T237I) in the NIDO domain showed definitive high-frequency hearing loss, arguing against the previously proposed genotype-phenotype correlation. It would not have been possible to ascertain this without whole-exome sequencing. Whole-exome sequencing and further validation excluded other genes as possible causes of hearing loss in the family SNUBH18, thereby leaving p.T237I of *TECTA* as the only candidate. Of course, it is possible that genes that fell within the low- or no-coverage region on whole-exome sequencing might harbor a causative variant. Regardless, the variant c.710 C>T (p.T237I) was considered pathogenic and causative of the phenotype of SNUBH18 for the following reasons. First, SIFT and Polyphen prediction software consistently suggested that this variant was “damaging” (Table 2). Second, this variant was undetected in 334 (54+280) ethnically matched Korean control subjects and in 1,000 genomes. Third, p.T237 is well-conserved among species. Last, the same variant (c.710 C>T [p.T237I]) was detected in another Korean family, segregating with similar high-frequency hearing loss in an autosomal dominant fashion (personal communication with Dr. Hong-Joon Park).

**Table 2:** Whole filtering process to identify a causative variant of deafness from SNUBH18.

Gene Symbol	Variant Type	Exome Sequencing				Sanger Sequencing					Control Group		Functional impact prediction of nonsynonymous SNPs				
		III-1†	II-4†	II-5	II-1	I-1†	III-3†	II-2	II-3	III-2	Cohort #1 (70)	Cohort #2 (210)	PhyloPscore	SIFT	Polyphen-2	Mutation Taster	Mutation Assessor
ARHGAP31	c.4211C>G	Het	Het	.	.	O		O	X				2.200	Y	Y	Y	Low
	p.T1404R missense													0.00			0.895
FAM214A	c.1064C>G	Het	Het	.	.	O			O				4.194	N/A	N/A	Y	N/A
	p.S355X nonsense																
GPRC5B	c.805G>A	Het	Het	.	.	O		X	O				4.071	Y	Y	Y	Medium
	p.D269N missense													0.01			2.685
ITCH	c.17C>T	Het	Het	.	.	X		X	X				1.938	Y	N	Y	Low
	p.S6L missense													0.01			1.590
MICAL3	c.74G>T	Het	Het	.	.	O	X	X	X	O	N.D.^‡^	N.D.^ ‡^	4.393	Y	Y	N	Low
	p.C25F missense													0.03			1.720
PCDP1	c.1751T>C	Het	Het	.	.	O	X	X	X	O	N.D.^‡^	N.D.^‡^	2.403	Y	Y	Y	Low
	p.I584T missense													0.01			1.445
TECTA	c.710C>T	Het	Het	.	.	O	O	X	X	X	N.D.^‡^	N.D.^‡^	4.045	Y	Y	N	Medium
	p.T237I													0.01			2.955
	missense																
TPRG1	c.721G>A	Het	Het	.	.	X			X				4.269	Y	Y	Y	Medium
	p.V241M missense													0.00			2.275
WDR12	c.959A>G	Het	Het	.	.	O		O	X				5.143	Y	Y	Y	Neutral
	p.H320R missense													0.00			0.675

† III-1, II-4, I-1 and III-3 in bold: Affected, ‡N.D.: not detected.

Hildebrand et al. (2001) observed that homozygous mutant mice with a targeted deletion of the ENT domain of the alpha-tectorin gene display severe elevation of the mid-frequency hearing threshold (5–20 kHz) [41], which supported their genotype-phenotype correlation. However, the mouse model likely reflects a null status of alpha-tectorin, not the effect of an autosomal dominant mutation, because heterozygous knockout mutant mice showed no phenotype [41]. A diverse genotype-phenotype correlation for the mutations may be located in the NIDO domain of *TECTA*, depending on the exact location in the domain. The NIDO domain of alpha-tectorin has been predicted to facilitate the assembly and modeling of the extracellular matrix of the tectorial membrane, interacting with laminin and type IV collagen [42]-[44]. Generation of a knock-in mouse model of the p.T237I mutation and evaluation of the mechanotransduction of mid and high frequencies may reveal the reason for this phenotypic difference between the previously reported mutations and the mutation discovered in this study in the same domain. Our result clearly shows that a diverse genotype-phenotype correlation can exist, even within the same domain of the protein, proposing a possible “variant to phenotype correlation” beyond “gene to phenotype” or “domain to phenotype” correlations. Verification of the various effects of the mutations on either the structure or the function of alpha-tectorin, as suggested by the different phenotypes, will further the understanding of the role of the tectorial membrane.

The tectorial membrane is formed during development, and tectorin is not usually replaced, based on the expression profile of tectorin in the developing cochlea [7], [8], [45]. This feature is compatible with the stable nature of most of the DFNA8/12 cases, except those in which a cysteine residue is mutated [12], [13], [16]. The progressive nature of hearing loss due to *TECTA* mutations appears to be correlated with a progressive loss of outer hair cells, as suggested by transient-evoked otoacoustic emission data from subjects carrying p.Cys1837Gly [16]. In contrast, this pathology has not been reported in mutant mice carrying a p.Tyr1870Cys mutation responsible for non-progressive stable hearing loss [41], [46]. It would have been very interesting to examine the distortion product-evoked otoacoustic or transient-evoked otoacoustic emission results from subjects II-4 and III-1, who carry p.T237I of the *TECTA* gene. However, these subjects refused the test, leaving the status of their outer hair cells unknown.

Although next-generation per-base sequencing costs have become relatively low, the cost of sequencing the entire human genomes remains high. Whole-genome sequencing is unnecessary for many diagnostic and research applications, because the protein-coding regions of genes (exons) constitute an estimated 1% of the genome but harbor 85% of the mutations, which have large effects on disease [23]. After evaluating whole-exome sequencing from four family members and performing subsequent Sanger sequencing validation in an additional five family members, we are almost confident that this *TECTA* variant is the causative variant. A mutation in a neighboring gene that fell within the no- or low-coverage region on the whole-exome sequence might account for the high-frequency hearing loss in this family, and p.T237I in *TECTA* may have coincidentally been in linkage disequilibrium with the true causative gene. However, a significant effect of p.T237I upon protein structure as predicted by homology modeling makes the hypothesis least likely.

We may find additional rare variants through further exome sequencing endeavors, but we are certain that the whole-exome sequencing covered at least 85% (depth ≥ 5) of the target enrichment region. Moreover, most rare nonpathogenic variants in exome sequences reside outside of coding sequences owing to the efficiency of microarray cross-hybridization. Furthermore, most disease-causing mutations are located in highly conserved regions, which exome sequencing is expected to cover.

## Conclusion

We identified a novel variant of *TECTA* and a phenotype that disputes a previously proposed genotype-phenotype correlation in the ENT domain of this gene. Because we acquired a thorough roadmap from whole-exome sequencing of five family members and undertook a subsequent segregation study using Sanger sequencing from an additional four members, we propose a novel genotype-phenotype correlation.

## Supporting Information

Figure S1 **The protein structure of the chain A, L-fucose isomerase (1FUI_A 499–568) fragment (A) p.T559 of 1FUI_A is equivalent to p.T237.** Yellow circle denotes p.T559. (B) View of the predicted protein structure from various angles when threonine is converted into isoleucine: yellow arrows denote the isoleucine residue, and a contact-map using the DISTIL utility (http://distill.ucd.ie/) indicates that the mutant residue is likely to be exposed to the surface, while the wildtype threonine residue (white arrow) is embedded in the structure (C).
(TIF)

Table S1 **Statistics of whole exome sequencing data for four individuals from SNUBH18.**
(DOCX)

Table S2 **Mutation profile of** ***TECTA*** **gene (located on chr11).**
(DOCX)

Table S3 **Pfam domain and Amino Acid Position.**
(DOCX)
