# Supplementary figures and images for: Whole-Exome Sequencing Identifies a Novel Genotype-Phenotype Correlation in the Entactin Domain of the Known Deafness Gene TECTA
Source: PLoS One. 2014 May 9;9(5):e97040. doi: 10.1371/journal.pone.0097040 (PMC4016231; doi:10.1371/journal.pone.0097040)

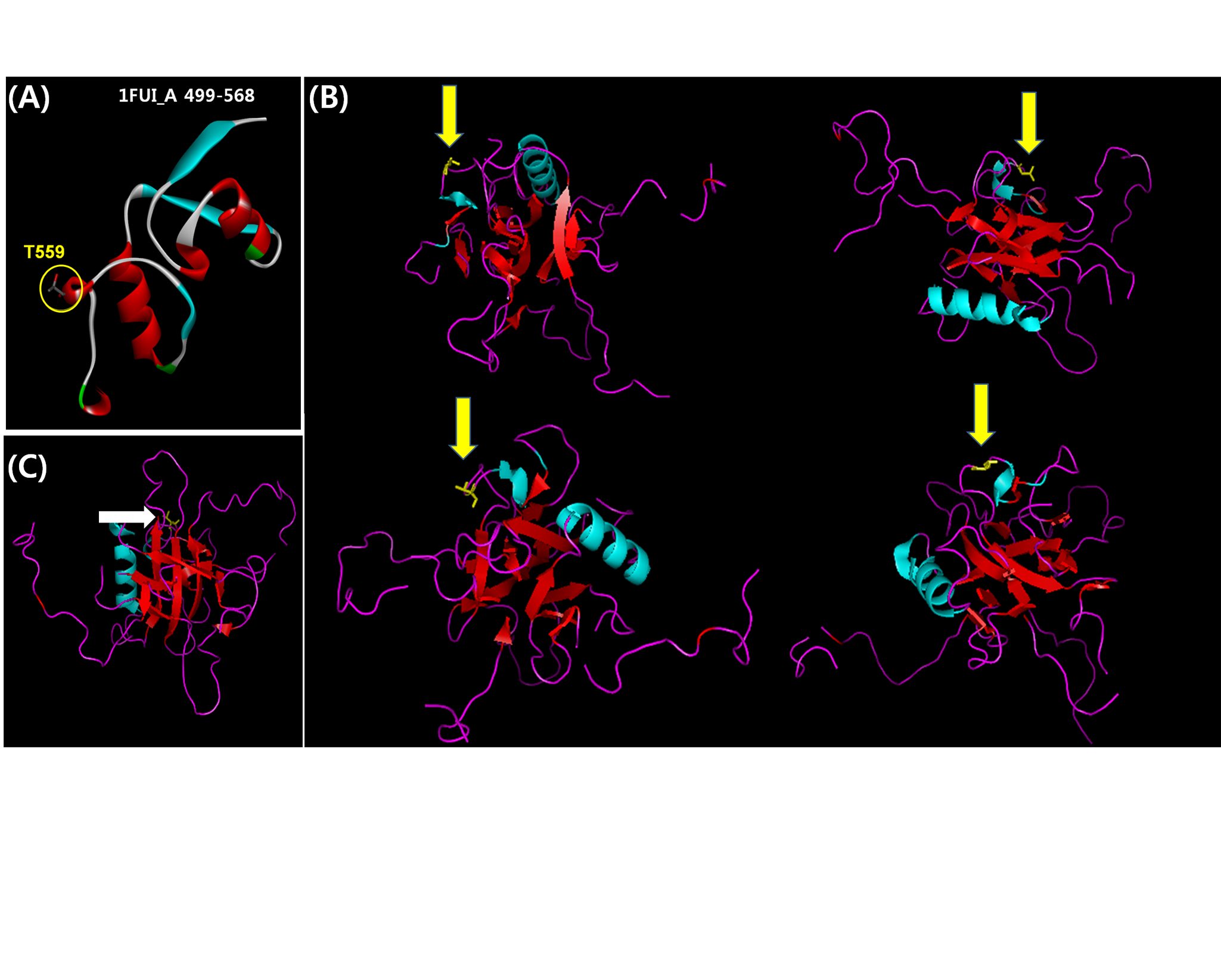

Supplement: Figure S1 — The protein structure of the chain A, L-fucose isomerase (1FUI_A 499–568) fragment (A) p.T559 of 1FUI_A is equivalent to p.T237. Yellow circle denotes p.T559. (B) View of the predicted protein structure from various angles when threonine is converted into isoleucine: yellow arrows denote the isoleucine residue, and a contact-map using the DISTIL utility (http://distill.ucd.ie/) indicates that the mutant residue is likely to be exposed to the surface, while the wildtype threonine residue (white arrow) is embedded in the structure (C). (TIF) [file pone.0097040.s001.tif]
