# Supplementary material for: Whole-Exome Sequencing Identifies a Novel Genotype-Phenotype Correlation in the Entactin Domain of the Known Deafness Gene TECTA
Source: PLoS One. 2014 May 9;9(5):e97040. doi: 10.1371/journal.pone.0097040 (PMC4016231; doi:10.1371/journal.pone.0097040)
Supplement: Table S1 — Statistics of whole exome sequencing data for four individuals from SNUBH18. (DOCX) [file pone.0097040.s002.docx]

**Table S1.** Statistics of whole exome sequencing data for four individuals from SNUBH18

|  | **Individuals** | **III-1** | **II-4** | **II-5** | **II-1** |
| --- | --- | --- | --- | --- | --- |
|  |  | **(Affected)** | **(Affected)** | **(Unaffected)** | **(Unaffected)** |
| **Mapping** | # reads | 54,744,038 | 69,091,300 | 32,507,684 | 41,681,496 |
|  | # mapped reads | 49,710,539 | 61,510,973 | 29,406,313 | 37,648,562 |
|  | mapping rate (%) | 90.8% | 89.0% | 90.5% | 90.3% |
|  | # duplicate removed reads | 40,769,185 | 53,578,727 | 26,298,247 | 32,152,881 |
|  | # on-target reads | 17,838,130 | 24,785,490 | 11,357,304 | 14,274,965 |
|  | on-target reads (%) | 43.8% | 46.3% | 43.2% | 44.4% |
|  | Mean depth | 32.58 | 45.35 | 20.72 | 26.12 |
|  | %_bases_above_1 | 96.9 | 98.9 | 96.1 | 96.5 |
|  | %_bases_above_5 | 89.9 | 96.0 | 85.0 | 87.6 |
|  | %_bases_above_10 | 81.1 | 92.0 | 70.5 | 76.4 |
|  | %_bases_above_20 | 62.8 | 80.8 | 42.4 | 53.5 |
| **SNP** | total | 47,267 | 47,137 | 44,557 | 45,798 |
|  | homozygous | 19,792 | 19,600 | 19,426 | 18,844 |
|  | heterozygous | 27,475 | 27,537 | 25,131 | 26,954 |
|  | transition | 33,607 | 33,583 | 31,593 | 32,597 |
|  | transversion | 13,660 | 13,554 | 12,964 | 13,201 |
|  | exon | 16,866 | 16,809 | 16,035 | 16,508 |
|  | CDS | 14,598 | 14,542 | 13,898 | 14,333 |
|  | utr5 | 1,050 | 1,066 | 982 | 1,017 |
|  | utr3 | 901 | 888 | 846 | 849 |
|  | intron | 27,193 | 27,126 | 25,499 | 26,140 |
|  | non_coding_exon | 1,581 | 1,593 | 1,549 | 1,529 |
|  | non_coding_intron | 3,199 | 3,180 | 3,152 | 3,072 |
|  | silent | 7,112 | 7,137 | 6,788 | 7,015 |
|  | missense | 6,517 | 6,440 | 6,157 | 6,359 |
|  | nonsense | 59 | 52 | 49 | 55 |
|  | readthrough | 8 | 8 | 6 | 8 |
|  | startcodon | 9 | 7 | 13 | 8 |
|  | splicing | 44 | 44 | 44 | 44 |
| **INDEL** | total | 3,888 | 3,847 | 3,648 | 3,782 |
|  | homozygous | 1,495 | 1,503 | 1,513 | 1,444 |
|  | heterozygous | 2,393 | 2,344 | 2,135 | 2,338 |
|  | exon | 471 | 454 | 421 | 459 |
|  | CDS | 255 | 246 | 233 | 252 |
|  | utr5 | 98 | 102 | 86 | 99 |
|  | utr3 | 100 | 91 | 90 | 96 |
|  | intron | 3,179 | 3,152 | 2,994 | 3,091 |
|  | non_coding_exon | 71 | 65 | 63 | 62 |
|  | non_coding_intron | 333 | 306 | 315 | 304 |
|  | inframe | 103 | 96 | 92 | 98 |
|  | frameshift | 61 | 64 | 53 | 65 |
|  | nonsense | 7 | 5 | 4 | 5 |
|  | junction | 39 | 36 | 37 | 36 |
|  | startcodon | 0 | 0 | 0 | 0 |
|  | splicing | 33 | 29 | 27 | 29 |
| **Variant** | NS/SS/I | 6,887 | 6,790 | 6,495 | 6,719 |
|  | and deleterious (phyloP, SIFT) | 873 | 856 | 819 | 830 |
|  | and not in dbSNP v138 | 55 | 48 | 49 | 49 |
|  | and not in in-house database* | 47 | 42 | 40 | 43 |
| **Gene** | NS/SS/I | 4,092 | 4,028 | 3,860 | 3,973 |
|  | and deleterious (phyloP, SIFT) | 735 | 724 | 686 | 693 |
|  | and not in dbSNP v138 | 54 | 47 | 48 | 48 |
|  | and not in in-house database* | 46 | 41 | 39 | 42 |

*In-house Database: An independent cohort from normal individuals
