# Supplementary material for: Whole-Exome Sequencing Identifies a Novel Genotype-Phenotype Correlation in the Entactin Domain of the Known Deafness Gene TECTA
Source: PLoS One. 2014 May 9;9(5):e97040. doi: 10.1371/journal.pone.0097040 (PMC4016231; doi:10.1371/journal.pone.0097040)
Supplement: Table S2 — Mutation profile of TECTA gene (located on chr11). (DOCX) [file pone.0097040.s003.docx]

**Table S2.** Mutation profile of *TECTA* gene (located on chr11)

| Variant  Type | dbSNP  (v.138) | III-1  (Affected) | II-4  (Affected) | II-5  (Unaffected) | II-1  (Unaffected) | In-house database |
| --- | --- | --- | --- | --- | --- | --- |
| intronic | **rs681311** |  | **Het** |  |  | **Yes** |
| intronic | **rs504626** |  | **Het** |  |  | **Yes** |
| c.710C>T  p.T237I  missense | **Novel** | **Het** | **Het** |  |  | **No** |
| c.1111A>G  p.R371G  missense | **rs612969** | **Het** |  | **Het** |  | **Yes** |
| c.1485A>G  p.A495A  silent | **rs536069** | **Het** |  |  | **Het** | **Yes** |
| c.2795T>C  p.V932A  missense | **rs520805** | **Het** |  |  |  | **Yes** |
| c.2805T>C  p.Y935Y  silent | **rs586473** | **Het** |  | **Hom** | **Het** | **Yes** |
| c.5171G>A  p.S1724N  missense | **rs526433** | **Hom** | **Hom** |  |  | **Yes** |
| intronic | **rs2186747** | **Het** |  | **Het** |  | **Yes** |
| c.5634C>T  p.S1878S  silent | **rs2155369** |  |  | **Het** |  | **Yes** |
| intronic | **rs543577** |  |  | **Hom** |  | **Yes** |
