# Supplementary material for: Whole-Exome Sequencing Identifies a Novel Genotype-Phenotype Correlation in the Entactin Domain of the Known Deafness Gene TECTA
Source: PLoS One. 2014 May 9;9(5):e97040. doi: 10.1371/journal.pone.0097040 (PMC4016231; doi:10.1371/journal.pone.0097040)
Supplement: Table S3 — Pfam domain and Amino Acid Position. (DOCX) [file pone.0097040.s004.docx]

**Table S3.** Pfam domain and Amino Acid Position

| Pfam | AA Position | Symbol | Pfam Domain Name |
| --- | --- | --- | --- |
| PF06119 | 163 – 251 | NIDO**^#^** | nidogen-like domain |
| PF00094 | 322 – 477  713 – 865  1100 – 1257  1487 - 1638 | VWD | von Willebrand factor type D domain |
| PF08742 | 520 – 591  907 – 981  1296 – 1368  1686 - 1758 | C8 | C8 domain (8 conserved cysteine residues) |
| PF01826 | 597 – 650  984 – 1036  1372 - 1425 | TIL | Trypsin Inhibitor like cysteine rich domain |
| PF00100 | 1805 – 2058 | ZP | Zona pellucida-like domain (ZP domain / ZP-like domain) |

**^#^**Current version of Pfam reorganized ENT (entactin-like domain) as NIDO domain and it has shorter domain length. ENT domain is located in 98 - 252.
